# Mesofluidic Devices for DNA-Programmed Combinatorial Chemistry

**DOI:** 10.1371/journal.pone.0032299

**Published:** 2012-03-29

**Authors:** Rebecca M. Weisinger, Robert J. Marinelli, S. Jarrett Wrenn, Pehr B. Harbury

**Affiliations:** 1 Department of Chemistry, Stanford University, Stanford, California, United States of America; 2 Department of Biochemistry, Stanford University, Stanford, California, United States of America; The Scripps Research Institute, United States of America

## Abstract

Hybrid combinatorial chemistry strategies that use DNA as an information-carrying medium are proving to be powerful tools for molecular discovery. In order to extend these efforts, we present a highly parallel format for DNA-programmed chemical library synthesis. The new format uses a standard microwell plate footprint and is compatible with commercially available automation technology. It can accommodate a wide variety of combinatorial synthetic schemes with up to 384 different building blocks per chemical step. We demonstrate that fluidic routing of DNA populations in the highly parallel format occurs with excellent specificity, and that chemistry on DNA arrayed into 384 well plates proceeds robustly, two requirements for the high-fidelity translation and efficient *in vitro* evolution of small molecules.

## Introduction

Natural evolution consists of iterated cycles of gene diversification, gene expression, functional selection and reproductive amplification. These cycles can be re-enacted in a test tube using populations of random biopolymer sequences as the genetic units. Functional selection is imposed by requiring individual molecules to bind to a target, or to catalyze coupling to an affinity handle, in order to survive. Remarkably, novel snippets of nucleic acid and protein with the selected functional property (binding or catalytic proficiency) emerge. The test-tube evolution paradigm can be extended to small-molecule genetic units through DNA-programmed combinatorial chemistry.[1], [2], [3], [4] Ribosomal translation is replaced with “chemical translation,” wherein a DNA gene sequence programs the chemical synthesis of a covalently attached small molecule.[5]–[6] DNA-programming enables the propagation and breeding of small-molecule populations over multiple generations.

By analogy to *in vitro* biopolymer evolution, it has been suggested that evolving small-molecule libraries of more than ten billion compounds for binding to a protein target should yield ligands with dissociation constants in the nanomolar range.[1] [7] There are a number of ways to construct chemical libraries of such high complexity. One strategy would be to create synthetic decamers from an alphabet of ∼10 chemical building blocks. This strategy produces high molecular-weight compounds that do not resemble small-molecule drugs, like those in the World Drug Index.[8] Alternatively, one could construct molecules in four steps using an alphabet of 384 distinct building blocks at each synthetic step. This large-alphabet strategy minimizes the molecular weight of the individual molecules that make up the population. In order to create a large-alphabet library using DNA-programmed combinatorial chemistry, some technical innovations are required.

**Figure 1:**
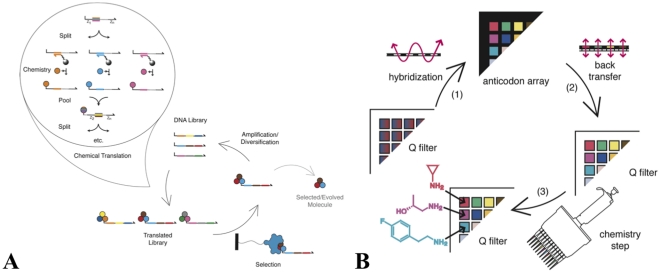
Small molecule evolution by DNA-programmed combinatorial chemistry. (**A**) A degenerate library of DNA “genes” is chemically translated into small molecule-DNA conjugates. The covalently attached small molecule corresponds to the structure encoded by the DNA gene. The small molecule-DNA conjugates are then selected for a desired trait, such as binding to a protein of interest. The encoding DNA is amplified and diversified. The cycle is iterated to yield selected/evolved small molecules with the desired trait. (**B**) One step of a highly parallel DNA-programmed chemical translation. (**1**) A degenerate DNA library (in which the identity at the first coding position is depicted by a range of colors) on an ion-exchange filter is split by hybridization to complementary oligonucleotides on an anticodon array. (**2**) The separated pools (with distinct colors representing isolated codon sequences) are transferred to a fresh anion-exchange chemistry array. (**3**) The encoded building blocks are incorporated by chemical coupling. These steps are repeated for each coding position until the DNA “gene” is fully translated.

Here, we report tools that facilitate the construction of highly complex libraries with the possibility for hundreds of diversity elements at each position. These tools build on a previously described approach to chemical translation that involves spatial partitioning of a DNA population by hybridization followed by spatially determined chemical coupling steps (Figure 1a).[6] A read of a single coding position is illustrated in Figure 1b. A degenerate library of single-stranded DNA genes is split by hybridization into different wells of a cassette holding 384 distinct oligonucleotide-conjugated resins, the “anticodon array.” Following hybridization, the DNA sequences are transferred in a one-to-one fashion onto a 384-feature anion-exchange array for execution of a chemistry step on solid-supported DNA. The solid support allows reactions to be driven to completion with excess reagents, and allows reactions to be performed under conditions that are incompatible with DNA hybridization and DNA solubility. After the chemical coupling step, the library is pooled and split again by hybridization at the next coding position. Additional reads are performed until all of the coding positions have been translated.

## Results

Our original implementation of DNA-programmed combinatorial chemistry used commercially available oligonucleotide synthesis columns to house the anticodon resins required for library splitting and the anion-exchange resins required for chemical synthesis steps.[9] This approach was inexpensive and convenient for libraries with small alphabets, but becomes unwieldy with large numbers of building blocks. Consequently, we set out to develop arrayed formats to facilitate the synthesis of large-alphabet libraries. We focused on planar substrates with a standard microplate footprint that could exploit the tools developed for high-throughput chemistry and biology, including multi-well plates, plate centrifuges, multi-channel pipetters, and pipetting robots.

**Figure 2:**
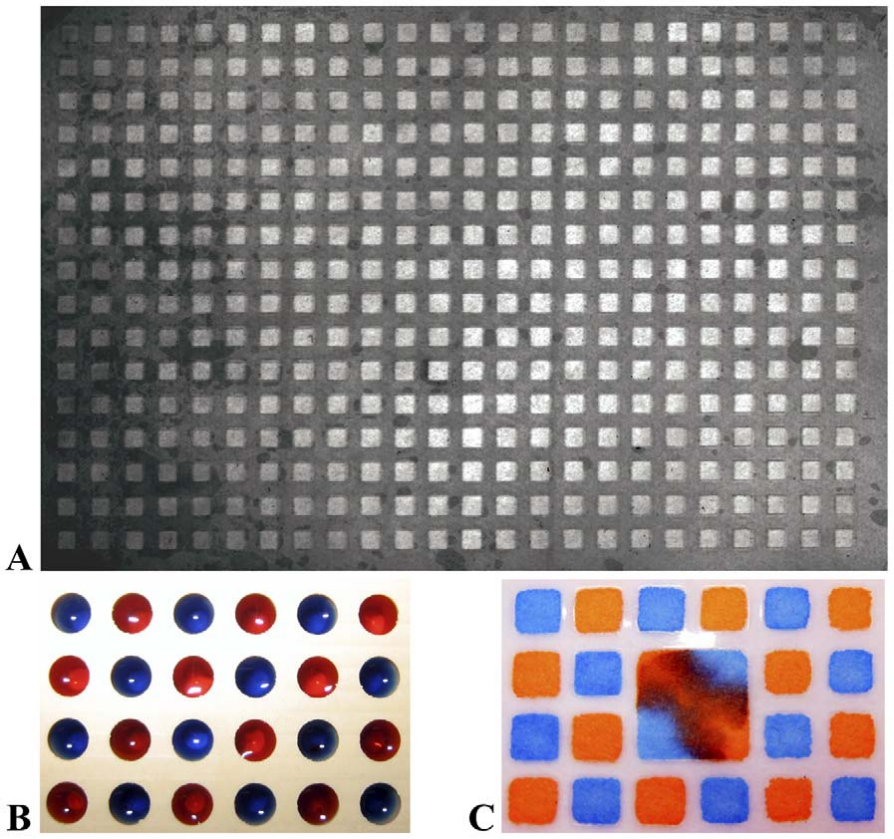
Anion-exchange chemistry arrays. (**A**) A cellulose filter patterned with photocurable fluoropolymer. (**B**) The filter clamped between the two plates of the chemistry apparatus. The wells are filled in a checkerboard pattern with solutions of Orange G and xylene cyanol. (**C**) After evacuating the dye solutions and washing on a vacuum manifold, the filter is removed from the chemistry apparatus. The dyes mix only in the central squares, which lack fluoropolymer barriers between neighboring wells.

We first created a chemistry array for carrying out reactions in parallel. Our efforts built on the SPOT synthesis literature in which cellulose paper is used as a stable support for the synthesis of covalently attached chemical libraries.[10] [11] To use cellulose supports for DNA-programmed combinatorial chemistry, the membrane must act as a strong anion-exchange material that can bind reversibly to DNA. We therefore derivatized the surface of the cellulose paper with quaternary amines using a process described by Genentech for the production of charged filtration membranes.[12] Typically, SPOT synthesis is performed on dense arrays of synthetic sites with no chemical or physical barriers between the sites, relying on small reagent quantities to prevent mixing of reactants at neighboring positions. Chemical transformations on solid support, however, often require the use of a reagent excess to push reactions to completion. Reagent excesses cause reactant mixing between spots on a cellulose array (Figure 2). We therefore investigated how to separate the reaction sites with a chemically resistant polymer. We solved the problem by imbedding a photo-curable fluoroelastomer[13] into the quaternary amine-modified cellulose membrane. Precise patterning was accomplished by using masks to photo-polymerize a fluoropolymer border around a field of open wells (Figure 2a) in an approach similar to the paper microzone plates reported by Carrilho and coworkers.[14] The chemically resistant gasketing prevents the contents of adjacent wells from mixing (Figure 2b and 2c).

**Figure 3:**
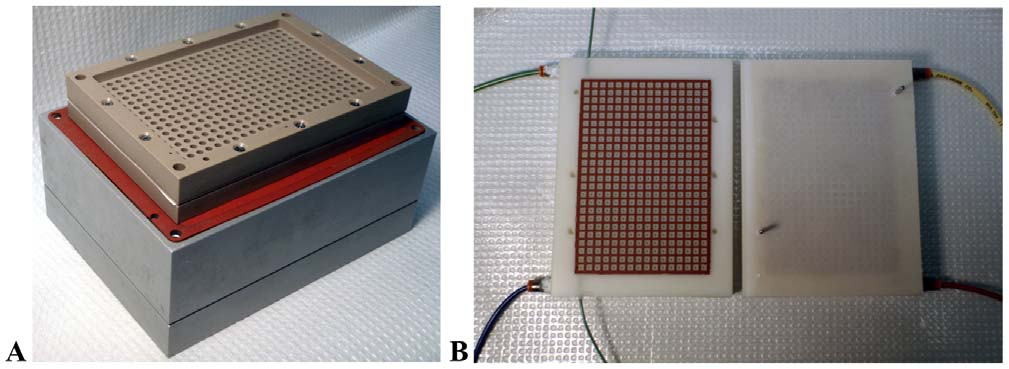
Mesofluidic devices for DNA-programmed synthesis. (**A**) The chemistry apparatus on top of a Qiagen multi-well plate manifold. The top half of the device has a large trough to hold fluid for wash steps, and the bottom half has a raised surface that acts as an array of drip directors to facilitate the collection of material. The interior surfaces of the plates are grooved to accommodate silicone gaskets. (**B**) The two halves of the mesofluidic pump prior to assembly. An anticodon array is loaded onto the right plate of the pump. Throughout the temperature cycle, the DNA library is circulated over all 384 features of the anticodon array. The thick colored tubing at the top and bottom of each plate delivers compressed air or vacuum to a subset of the diaphragm features in the plate. The thin green tubing at the top and bottom of the left plate is the liquid inlet/outlet.

**Figure 4:**
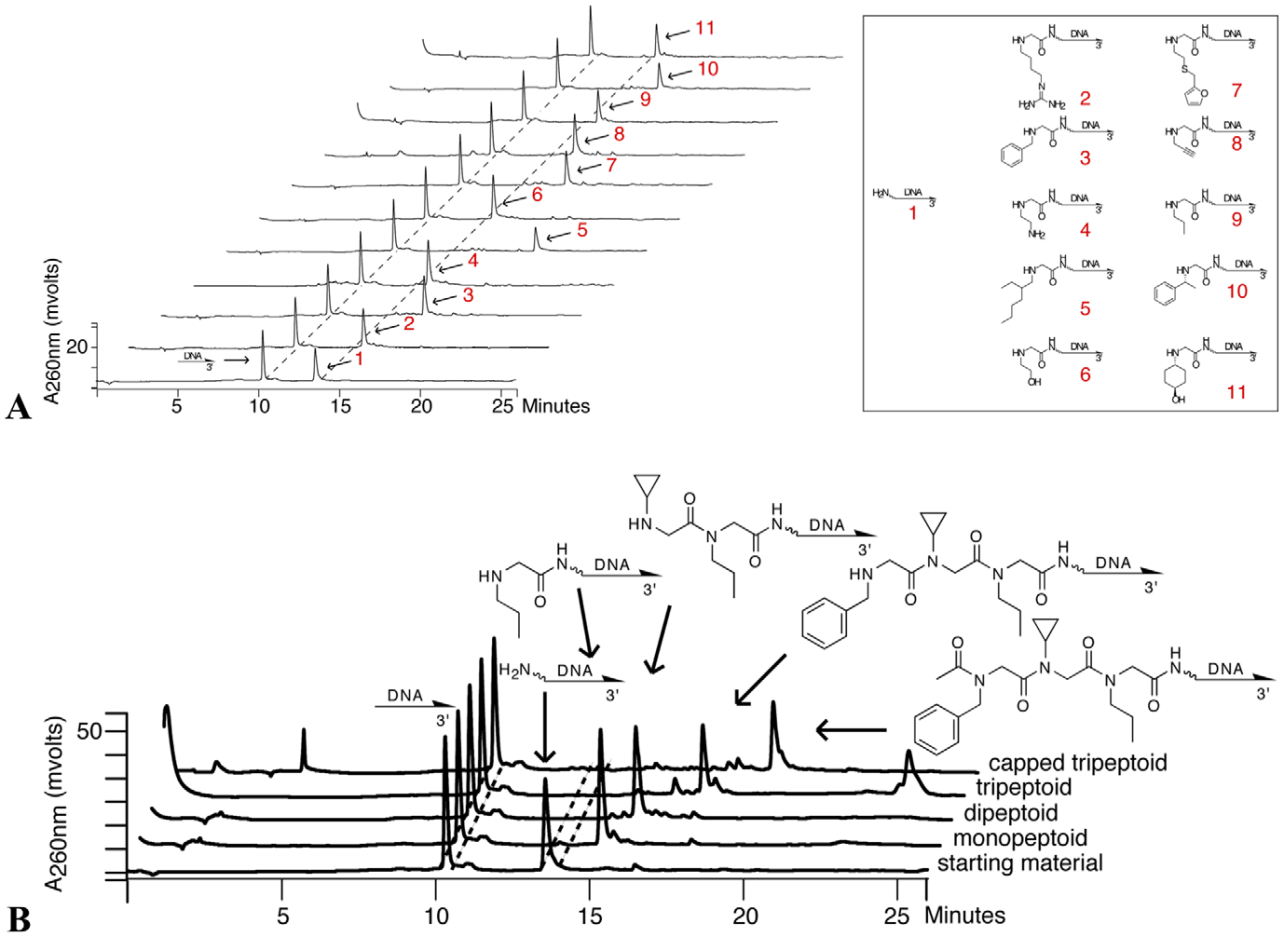
Parallel peptoid synthesis reactions on anion-exchange arrays. (**A**) A control 10-mer and a 5′ aminated 20-mer oligonucleotide were loaded in adjacent wells of a gasketed Q cellulose chemistry array and subjected to a chloroacetylation step followed by an alkylation step with one of 10 primary amines. After elution, the products were analyzed by reverse-phase HPLC. The peaks corresponding to the peptoid products were isolated, digested with P1 nuclease, and analyzed by LC-MS to confirm the identity of the product. The masses obtained by LC-MS are listed in the supporting materials (Table S1). (**B**) A control 10-mer and a 20-mer oligonucleotide with a 5′ primary amine were loaded in adjacent wells of a gasketed Q cellulose chemistry array (starting material) and subjected to one (monopeptoid), two (dipeptoid), or three (tripeptoid) peptoid coupling steps. Additionally, a synthesis consisting of three peptoid coupling steps and a subsequent acetylation step (capped tripeptoid) was performed. After elution, the products were analyzed by reverse-phase HPLC. The peak for the capped tripeptoid synthesis was isolated, digested with nuclease P1, and submitted for analysis by LC-MS. The identity of the major peak (approximately 88% yield) was confirmed ([M-H]^−^ observed, 898.99; expected, 898.47).

To create reagent reservoirs above the wells of the chemistry array, we designed plates with 384 through holes and grooves to receive in-plane rubber gaskets (Figure 3a). When sealed on either side of the array, the plates form 384 isolated reaction vessels, which allow for different reactions to occur in adjacent wells without measurable bleed over (Figure 4a). Because the arrays have the same footprint as commercial 384-well plates, wash steps can be performed using a standard microplate vacuum manifold.

**Figure 5:**
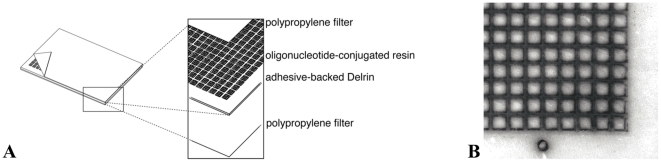
Construction of an anticodon array. (A) Oblique and exploded views of an anticodon array. The arrays are constructed by sealing one side of an adhesive-backed, lasercut Delrin sheet with a polypropylene filter, filling the resulting wells with oligonucleotide-conjugated resin, and then sealing the opposite side of the array with a second polypropylene filter. (B) Photograph of the lower right corner of an anticodon array.

Our DNA-programmed combinatorial chemistry approach also requires a means to split DNA by hybridization into 384 different sub-pools arranged in a planar format. One strategy would be to immobilize oligonucleotides onto a filter, but extensive studies with filter immobilization led us to the conclusion that filters cannot provide sufficient hybridization capacity. As an alternative, we used oligonucleotide-derivatized Sepharose resins housed in an array of 384 microcolumns. The microcolumns were constructed by laser cutting 3 mm square holes into a 380 µm-thick Delrin sheet, and then adhering polypropylene filters to either side. The polypropylene filters act as frits to hold Sepharose resin within each 3 mm wide and 380 µm long “column housing” (Figure 5). We call these planar structures “anticodon arrays” because each feature of the array contains a unique DNA sequence complementary to one of the codons used for DNA programming. The microcolumns hold microliter quantities of resin and remain stable to multiple rounds of hybridization and denaturation.

During a programmed chemical step, a DNA library is partitioned by hybridization to the 384 features of the anticodon array. The entire DNA library must be distributed over all of the features on a short timescale (defined by the rate of change of the hybridization temperature). Manually passing the library through every feature of the anticodon array by vacuum filtration or by centrifugation would require at least 384 manipulations of the pooled material and was deemed too laborious. Alternatively, one could use passive mixing or capillary action to route fluid.[15] Passive hybridization proved to be slow and provided many surfaces for the nonspecific binding of library material. Ideally, the library would be pumped cyclically over all of the features of the anticodon array, with minimal surface area and dead volume. If the system were heated and then cooled slowly, it would provide multiple passes of the whole library through each microcolumn of the anticodon array in the temperature window over which hybridization occurs.

**Figure 6:**
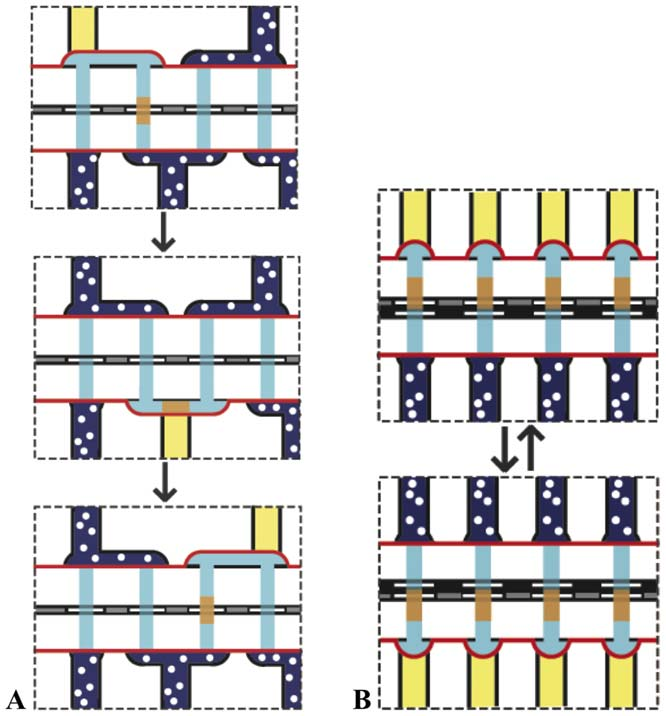
Schematic of hybridization pump and mesofluidic Southern blotter. (**A**) The hybridization pump is shown in cross-section with the plane of the elastomeric diaphragm colored red. One volume element of fluid is depicted as an orange rectangle. Alternating application of 15 psi of air pressure (blue with white spots) and house vacuum (yellow) directs the path of fluid flow. (**B**) A cross-section of the mesofluidic Southern blotter illustrating the movement of liquid through the stacked anion-exchange chemistry array and the anticodon array as air and vacuum are applied.

To realize the latter scheme, we constructed a mesofluidic pump (Figure 3b) that pushes liquid through the 384 features of an anticodon array in a serpentine path. As outlined in Figure 6a, application of alternating compressed air and vacuum to elastic diaphragms above and below the array produces liquid flow. This mesofluidic device is a positive displacement pump, and it operates by the same principles as the peristaltic micro-pumps used by the microfluidics community.[16]

Following spatial partitioning by hybridization, the physically separated sub-pools of DNA must be transferred from the anticodon array onto a cellulose chemistry array. To perform this transfer efficiently and conveniently, we constructed a mesofluidic Southern blotter (Figure 6). The blotter consists of two plates, each housing 384 isolated liquid columns loaded with a 10 mM sodium hydroxide solution. A chemistry array and anticodon array are clamped between the two plates. Application of alternating compressed air and vacuum to elastic diaphragms at the top and bottom of each column forces liquid to move up and down through the stacked arrays. Perfusion of the anticodon array with a denaturing solution causes release of DNA, and advection of the liquid column carries the DNA to the anion-exchange chemistry array where it rebinds. Isolation of the 384 independent liquid columns ensures a faithful one-to-one transfer of DNA between the features of the two arrays.

Collectively, the procedures described above complete one full read of a DNA-programmed synthesis. For a multi-step combinatorial library synthesis, the cycle of splitting, blotting and chemical modification is repeated multiple times.

### Fidelity of DNA-programmed chemistry

The fidelity of DNA-programmed combinatorial chemistry is dependent on two things: the accuracy with which the DNA population is split into spatially separated sub-pools, and the efficiency of the subsequent sub-pool specific chemistry steps. To characterize the arrays and mesofluidic devices described above, we measured these two parameters.

**Figure 7:**
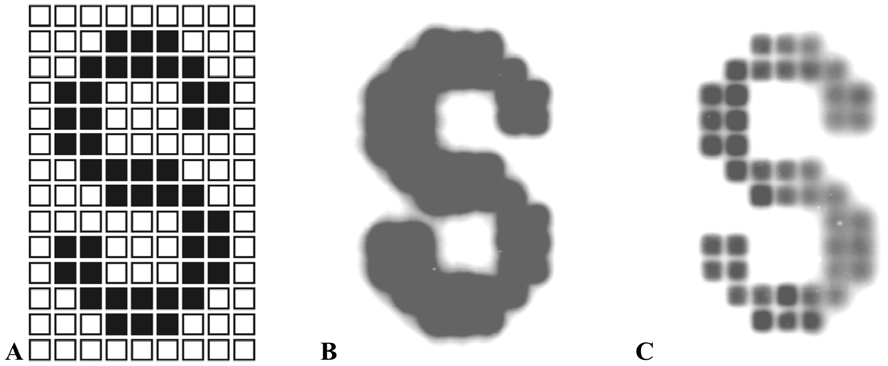
Patterned hybridization on an anticodon array and subsequent transfer to a chemistry array. (**A**) An anticodon array was assembled with two distinct oligonucleotide-coupled resins in the pattern of the letter S as shown in this schematic. (**B**) The anticodon array was then probed using a radiolabelled 40-mer complementary to one of the two oligonucleotides. Hybridization was performed with the mesofluidic pump. The array was imaged on a phosphor screen. (**C**) The hybridized 40-mer was transferred from the anticodon array onto an anion-exchange chemistry array using the mesofluidic Southern blotter, and imaged on a phosphor screen. Image C has finer resolution than image B because the chemistry array is much thinner than the anticodon array.

First, we tested the accuracy of the DNA splitting onto anticodon arrays. For these experiments, an anticodon array was constructed using two different oligonucleotide-conjugated resins. One of the two resins was added to wells that form the shape of the letter S, and the second resin was used to fill the remaining wells as shown in the schematic in Figure 7a. Radiolabelled 40-mers complementary to the resin used to define the “S” were hybridized to the array with the positive displacement pump for one hour at 45°C (the pump displaces the dead volume of the system in approximately five minutes). After hybridization, the anticodon array was imaged (Figure 7b), and the flowthrough was analyzed using a scintillation counter. More than than 95 percent of the radioactivity had bound. The 40-mers on the anticodon array were then transferred to an anion-exchange chemistry array using the mesofluidic Southern blotter, and the arrays were imaged again (Figure 7c). The labeled DNA partitioned to the “S” wells as expected, and the intensity of signal on the spots complementary to the 40-mer probe oligonucleotide were at least 29-fold higher than the background on adjacent non-complementary wells, showing that these devices can accurately divide DNA populations by sequence identity into spatially patterned sub-pools.

Second, we verified by HPLC that DNA (a 20-mer oligonucleotide with a 5′ primary amine modification) could be bound, chemically modified, and eluted from the anion-exchange chemistry array. Within the accuracy of our measurements, the binding and elution steps were quantitative. In adjacent wells, we performed one to four steps of a chemical synthesis employing peptoid chemistry. The final product of the four-step procedure was an acylated tripeptoid. Peptoid chemistry was used as a test case because it is one of several synthetic schemes that are versatile enough to accommodate hundreds to thousands of monomers in a multistep synthesis. The four-step, microwave-assisted synthesis in Figure 4b, which produces a representative member of a library of capped tripeptoids, proceeds with greater than 95 percent recovery of the DNA and 88 percent conversion to the desired product, a yield comparable to previously reported syntheses of peptoid-DNA conjugates in a column format.[1]

## Discussion

The specific DNA hybridization and efficient chemical conversion facilitated by the arrays and mesofluidic devices reported here constitute a high-fidelity system for DNA-programmed combinatorial chemistry. The highly parallel format should prove to be valuable in accessing large libraries with hundreds of diversity elements at each step of the synthesis. In future implementations, this format could easily be adapted for use with 1536-well plates. The microplate devices should facilitate the synthesis of small-molecule libraries with complexities comparable to those of the biopolymer libraries used for *in vitro* evolution.

The system described here and our previous realization of DNA-programmed combinatorial chemistry are limited in the amount of material that can be effectively synthesized; both yield roughly 50 picomoles of DNA-small molecule conjugate corresponding to ∼10^13^ molecules. As increasingly diverse DNA populations are translated, 50 picomoles will not adequately sample all of the library members. A system that could translate orders of magnitude more material would provide better coverage, and could potentially allow for larger fold-enrichments of fit molecules per generation.

A major challenge for DNA-programmed and DNA-encoded combinatorial chemistry technologies is the development of combinatorial synthetic schemes that accommodate hundreds to thousands of diversity elements at each step. Peptoid submonomer synthesis is one scheme that can achieve this building block complexity. Another is the sequential functionalization of triazine scaffolds with nucleophilic substituents, including Fmoc-amino acid building blocks.[2] These examples represent a small subset of the structures available to medicinal chemists. Combinatorial schemes that exploit other reagent classes or scaffolds would increase the region of chemical space that DNA-programmed chemical libraries can explore. For these reasons, efforts to expand DNA-compatible combinatorial chemistry will be extremely valuable.

Finally, routing of DNA populations can facilitate multiplexed selections of affinity reagents on a proteome-wide scale. For example, the microplate hybridization device could be used to divide bar-coded aptamer or mRNA display libraries for selections against 384 different targets. If the selected genetic material were pooled after selection into a common transcription/translation mixture, and then split by hybridization prior to the next selection round, the laborious library preparation process would be vastly simplified.

The recent successes of DNA-encoded combinatorial chemistry and its development and utilization in both academic and industrial settings point to a future in which these techniques will become an increasingly important part of the lead identification and drug optimization process. The tools we report will be useful in those efforts as they represent one straightforward and robust option for the programmed synthesis of diverse chemical libraries.

## Materials and Methods

### Materials

Chemicals and solvents were purchased from Acros (Geel, Belgium), Alfa Aesar (Ward Hill, MA, USA), Novabiochem (La Jolla, CA, USA), Oakwood Chemical (West Columbia, SC, USA), Sigma-Aldrich (St. Louis, MO, USA), TCI America (Portland, OR, USA), VWR International (West Chester, PA, USA), or from the supplier indicated.

5′ pentynyl oligonucleotides used for hybridization were purchased from Bioneer (Alameda, CA, USA). All other oligonucleotides, including sequences with reactive primary amine functionalities (Glen Research, Sterling, VA, USA; 5′-Amino Modifier C12 and Spacer Phosphoramidite with 5′-Amino Modifier 5), were purchased from the Stanford PAN Facility (Stanford, CA, USA).

### Equipment

Small parts and bulk materials (as described in the parts list in Supporting Information S1) were purchased from McMaster-Carr (Aurora, OH, USA) and Fisher Scientific. The ultraviolet flood lamp (#38100) used to cure the fluoropolymer gasketing material was purchased from Dymax (Torrington, CT, USA). Microwave-assisted chemical couplings were performed in a Panasonic NN-H965WFX 1250 W microwave. The pressure/vacuum manifold (M5A-0404-10) and valves (V3A-C231-AE1 and V3A-C231-BE1) were purchased from Mead USA (Chicago, IL, USA). The Stamp PLC (30064) and BASIC Stamp 2 module (BS2-IC) were purchased from Parallax (Rocklin, CA, USA). The laser cutter used to produce arrays and gaskets was a Legend 36EXT from Epilog Laser (Golden, CO, USA). The components for the routing devices were machined by Enviro-Tech (Boise, ID, USA), Patai Quality Machining (Santa Clara, CA, USA), and CCT Plastics (Grapevine, TX, USA). Engineering drawings are provided for these components in Figures S1, S2, S3, S4, S5, S6, S7, S8, S9, and S10). All 384-well vacuum filtration procedures were performed using a vacuum manifold (#9014579) from Qiagen (Venlo, Netherlands).

### General methods

HPLC analysis of peptoid reactions was performed on a Microsorb reverse-phase C18 analytical column (Varian; Palo Alto, CA, USA) heated to 50°C and monitored at 260 and 280 nm using a UV detector (Spectra Focus, Spectra-Physics; Irvine, CA, USA). Linear gradients between 100 mM triethylammonium acetate pH 5.5 and 100 mM triethylammonium acetate pH 5.5, 90 percent acetonitrile were used. DNA-peptoid conjugates were digested with P1 nuclease and analyzed on a Micromass ZQ LC-MS at the Vincent Coates Foundation Mass Spectrometry Laboratory (Stanford, CA, USA).

### Patterned quaternary ammonium **(**Q**)** cellulose

Rectangles measuring 11 by 15 cm were cut from sheets of cellulose filter paper (Whatman 542). Each rectangle was incubated in 15 mL of 1 M (3-bromopropyl) trimethylammonium bromide and 0.1 M sodium hydroxide at 37°C for 16 to 18 hours. Following derivatization, the filters were washed with water and acetone, dried, and flattened.

The rectangular cellulose filters were then patterned using photolithography. Each filter was immersed in 5 mL of a solvent-resistant photocurable liquid fluoropolymer, synthesized as described in reference 14 with the following modifications: Fluorolink D4000 (Solvay Solexis) was substituted for ZDOL, dichloropentafluoropropane (DCPFP) (SynQuest; Alachua, FL, USA) was substituted for Freon 113, and a silica plug was used to purify the polymer instead of an alumina column. The polymer-impregnated filter was placed between two masks of transparency film laser-printed with the negative image of a 384-well array, and the fluorinated material was polymerized by exposure to a UV flood lamp for approximately 20 s on each side. The unpolymerized material was removed by washing with DCPFP and acetone.

### Generation of azido-Sepharose

An amino azide PEG400 linker was synthesized following reference [17]. The linker (20 µmol) in 1 mL 200 mM diisopropylethylamine in N,N-dimethylformamide (DMF) was coupled to 100 mg NHS-activated Sepharose that had been previously washed with DMF. The reaction was incubated overnight at room temperature, washed with DMF, and incubated with 1 mL of 1 M ethanolamine in DMF for twelve hours at room temperature to cap unreacted sites. The resin was then washed with DMF and water and stored at 4°C.

### Synthesis of anticodon resin

A 5′ alkyne-modified phosphoramidite was prepared as previously reported by Duckworth and coworkers,[18] and two 20-mer oligonucleotides with the sequences 5 ′- GTGATTAAGTCTGCTTCGGC-3′ and 5 ′-CCCAGTGCTGACATCTATGA-3′ were synthesized by Bioneer (Alameda, CA, USA) using that material. 47 µl of 860 µM Cu(I) tris-(benzyltriazolylmethyl)amine (TBTA)[19] in DMSO were added to 54 µl of an aqueous solution of 20 µM crude alkyne-terminated oligonucleotide and 1 mM sodium ascorbate. The solution was incubated with the azide substrate for thirty minutes at room temperature. The remaining azide groups were capped by repeating the reaction with 1 M propargyl alcohol in place of the oligonucleotide.

### Anticodon arrays

Six inch segments were cut from a 0.015″ thick and 4″ wide strip of Delrin. Each piece was coated on both sides with double-sided tape (Scapa 702 Double Coated Silicone Tape). A 16 by 24 array of 3 mm-square holes on 4.5 mm centers was created using a laser cutter. To form a reservoir for the oligonucleotide-conjugated resin, the tape liner on one side of the array was removed, and a polypropylene filter with a 10 µm pore size (#60342) from Pall Corporation (Port Washington, NY, USA) was adhered to the adhesive surface. The array was inverted, the tape liner was removed from the opposite face, and the wells were filled with 10 µL of a 50∶50 water∶resin slurry. Excess fluid was removed using a vacuum manifold, and a second polypropylene filter was used to seal the array.

### Hybridization

The displacement pump was assembled so that an anticodon array was sandwiched between the internal plates, and the entire apparatus was clamped between two aluminum plates. The inlet and outlet tubes were connected to a buffer reservoir containing a radiolabeled 40-mer oligonucleotide (5′-ATGGTATCAAGCTTGCCAC AGCCGAAGCAGACTTAATCAC-3′). Air and vacuum pressure were used to pump buffer cyclically through the 384 features of the anticodon array. The application of air and vacuum was alternated using a valve array controlled by a BASIC stamp processor. A circuit diagram for the valve set-up (Figure S11) and the BASIC stamp programs can be found in the Supporting Information S1. After one hour in a water bath at 70°C, the temperature was lowered to 45°C for one hour and then to room temperature. Finally, the array was washed with 5 mL of hybridization buffer, and the system was disassembled. The array was incubated with a storage phosphor screen (Molecular Dynamics; Sunnyvale, CA, USA) for one hour, and the screen was imaged using a Typhoon 9400 (General Electric; Fairfield, CT, USA).

### DNA transfer

The two halves of the mesofluidic Southern blotter were filled with transfer buffer (10 mM sodium hydroxide, 0.005% Triton X-100, 1 mM EDTA). An anticodon array and a fresh chemistry array were stacked on one plate, then covered with the second plate, and secured with an aluminum clamp. The device was submerged in a water bath heated to 80°C. The air and vacuum lines were opened to approximately 10 psi of air pressure and house vacuum and connected to a valve array that alternated air and vacuum on each side of the arrays. After one hour, the assembly was removed from the water bath, unclamped, and the arrays were removed. Both the anticodon array and the ion-exchange array were incubated with a storage phosphor screen for five minutes. The screen was imaged as above.

### Peptoid coupling

The chemistry array with bound, 5′ amino modified oligonucleotides was secured between two gasketed plates used for chemistry. The array was then washed with 40 mL of methanol using a vacuum manifold. 40 µL of 150 mM 4-(4,6-dimethoxy-1,3,5-triazin-2-yl)-4-methylmorpholinium chloride and 100 mM sodium chloroacetate in distilled methanol were pipetted into each well and the reaction was incubated for 10 min at room temperature. The solvent was removed using a vacuum manifold, the array was washed with distilled methanol, and the acylation step was repeated twice more. After the third acylation step, the array was washed with 40 mL of 1 M propylamine in methanol and 40 mL of DMSO. Each well of the chemistry array was subsequently incubated with 40 µL of a 2 M solution of a primary amine in DMSO, and the entire assembly was microwaved for 13 s at 100% power six times over 30 min. The reactions were allowed to cool for approximately 5 min after each microwave step. Following alkylation, the arrays were washed with 40 mL each of DMSO and water. Finally, the chemistry plates were disassembled, and the DNA was eluted using Elute Buffer (50 mM Tris pH 8, 1.5 M NaCl, 0.005% Triton X-100).

## Supporting Information

Figure S1 **Engineering drawing of internal plate for backtransfer device.**
(TIFF)

Figure S2 **Engineering drawing of outer plate for backtransfer device.**
(TIFF)

Figure S3 **Engineering drawing of internal plate for backtransfer device.**
(TIFF)

Figure S4 **Engineering drawing of outer plate for backtransfer device.**
(TIFF)

Figure S5 **Engineering drawing of bottom plate for chemistry device.**
(TIFF)

Figure S6 **Engineering drawing of top plate for chemistry device.**
(TIFF)

Figure S7 **Engineering drawing of internal plate for mesofluidic pump.**
(TIFF)

Figure S8 **Engineering drawing of outer plate for mesofluidic pump.**
(TIFF)

Figure S9 **Engineering drawing of internal plate for mesofluidic pump.**
(TIFF)

Figure S10 **Engineering drawing of outer plate for mesofluidic pump.**
(TIFF)

Figure S11 **Circuit diagram for Stamp PLC.**
(TIFF)

Table S1 **Masses of peptoid-DNA conjugates in** **Figure 2b** **following digestion with nuclease P1.**
(DOCX)

Supporting Information S1 **Parts list and BASIC stamp programs for mesofluidic devices.**
(DOC)
