# Supplementary figures and images for: Mesofluidic Devices for DNA-Programmed Combinatorial Chemistry
Source: PLoS One. 2012 Mar 29;7(3):e32299. doi: 10.1371/journal.pone.0032299 (PMC3315586; doi:10.1371/journal.pone.0032299)

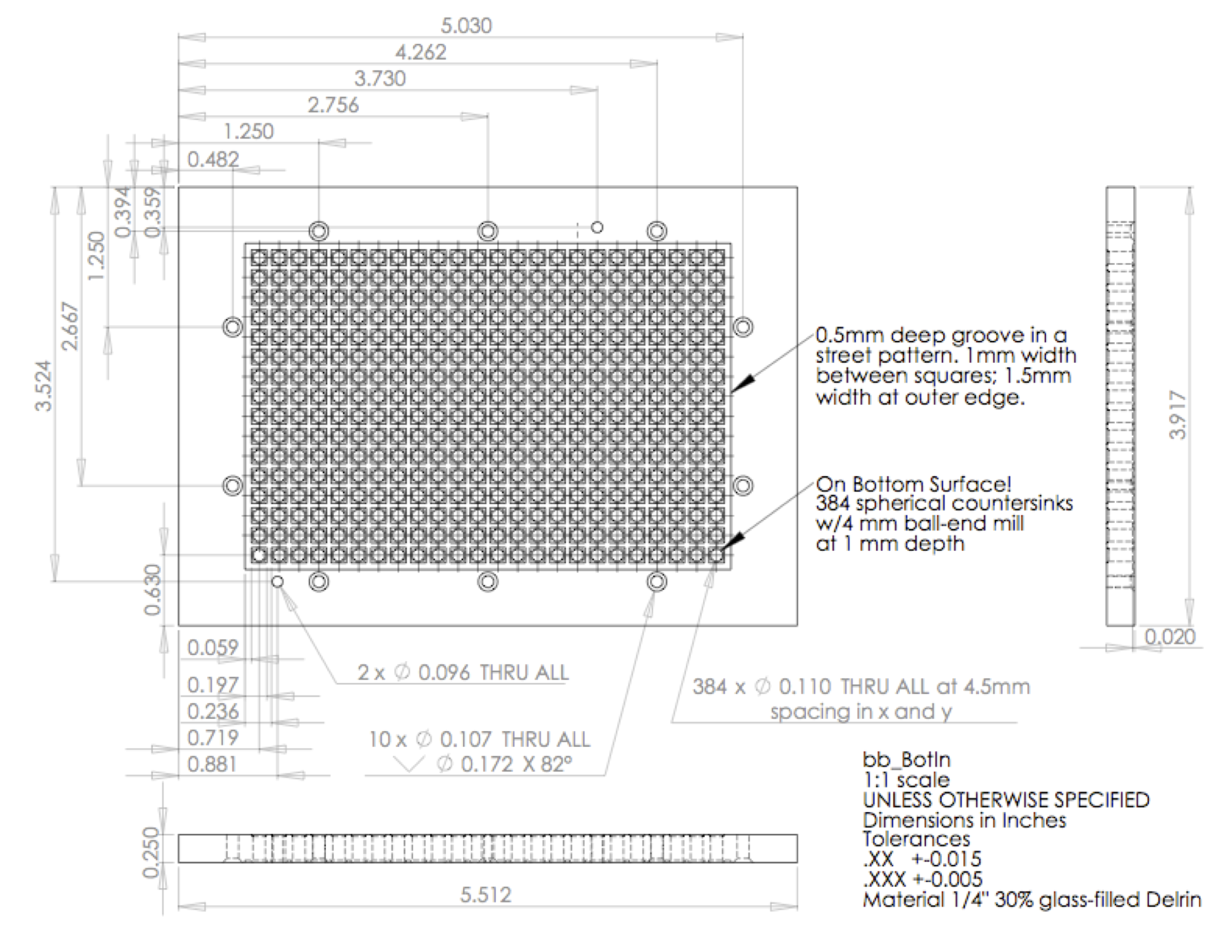

Supplement: Figure S1 — Engineering drawing of internal plate for backtransfer device. (TIFF) [file pone.0032299.s001.tif]

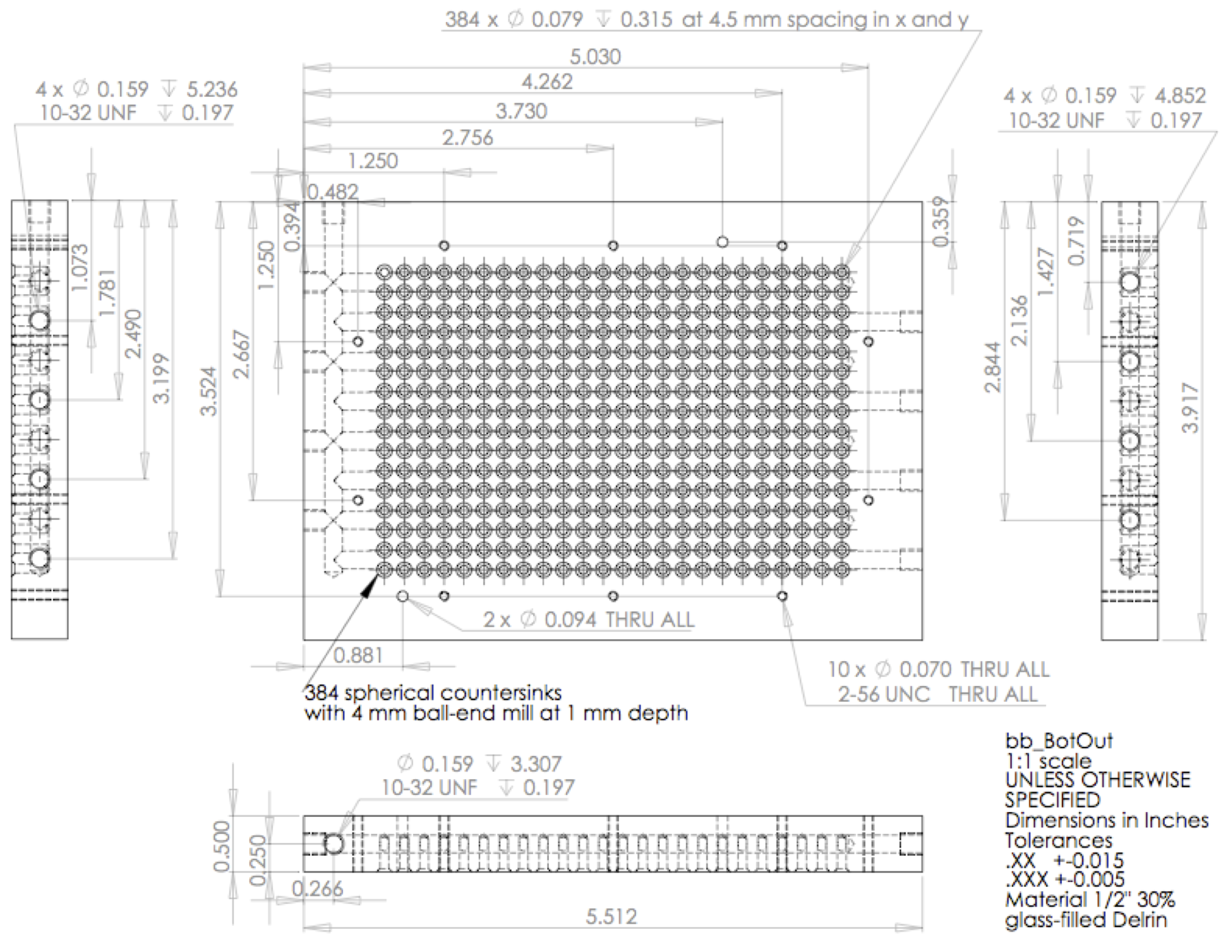

Supplement: Figure S2 — Engineering drawing of outer plate for backtransfer device. (TIFF) [file pone.0032299.s002.tif]

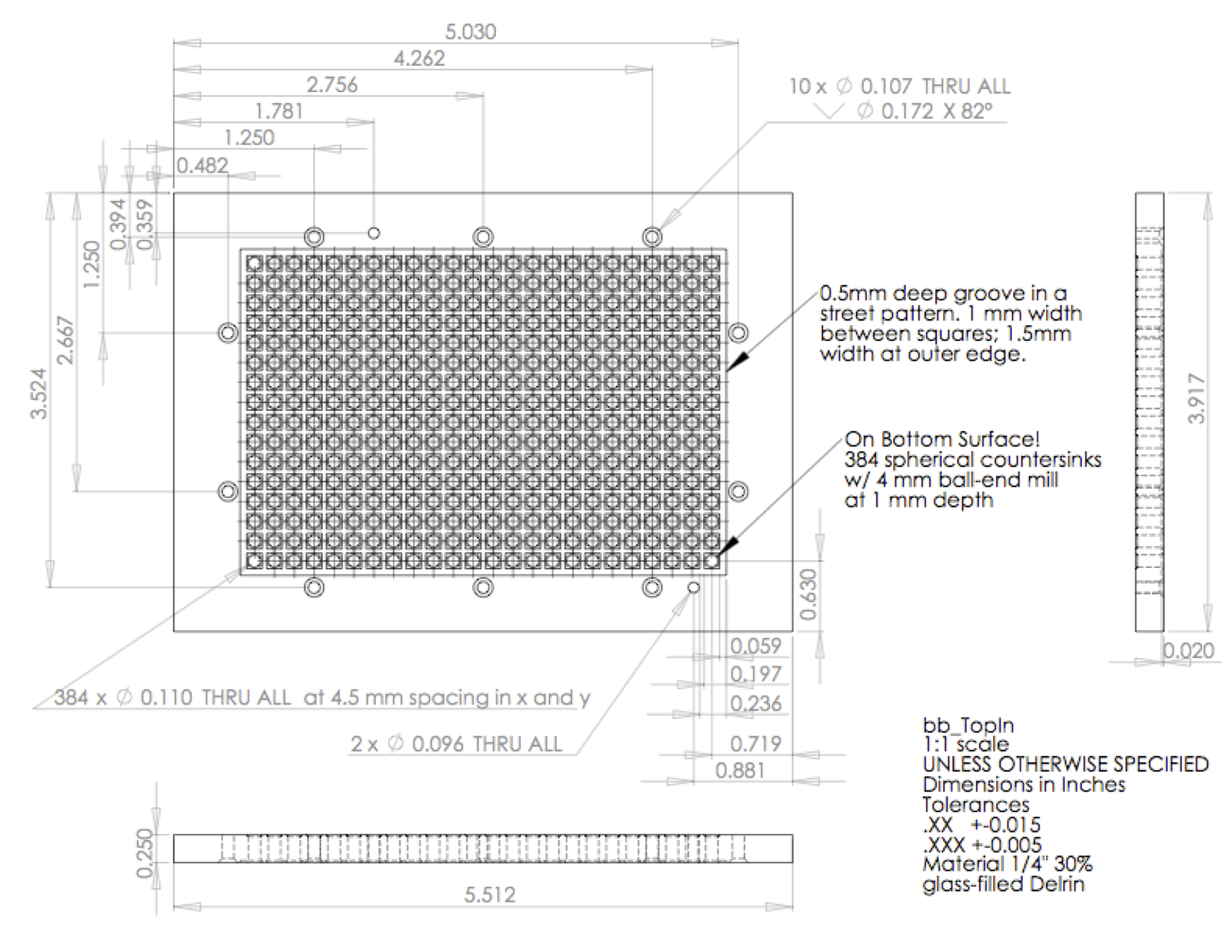

Supplement: Figure S3 — Engineering drawing of internal plate for backtransfer device. (TIFF) [file pone.0032299.s003.tif]

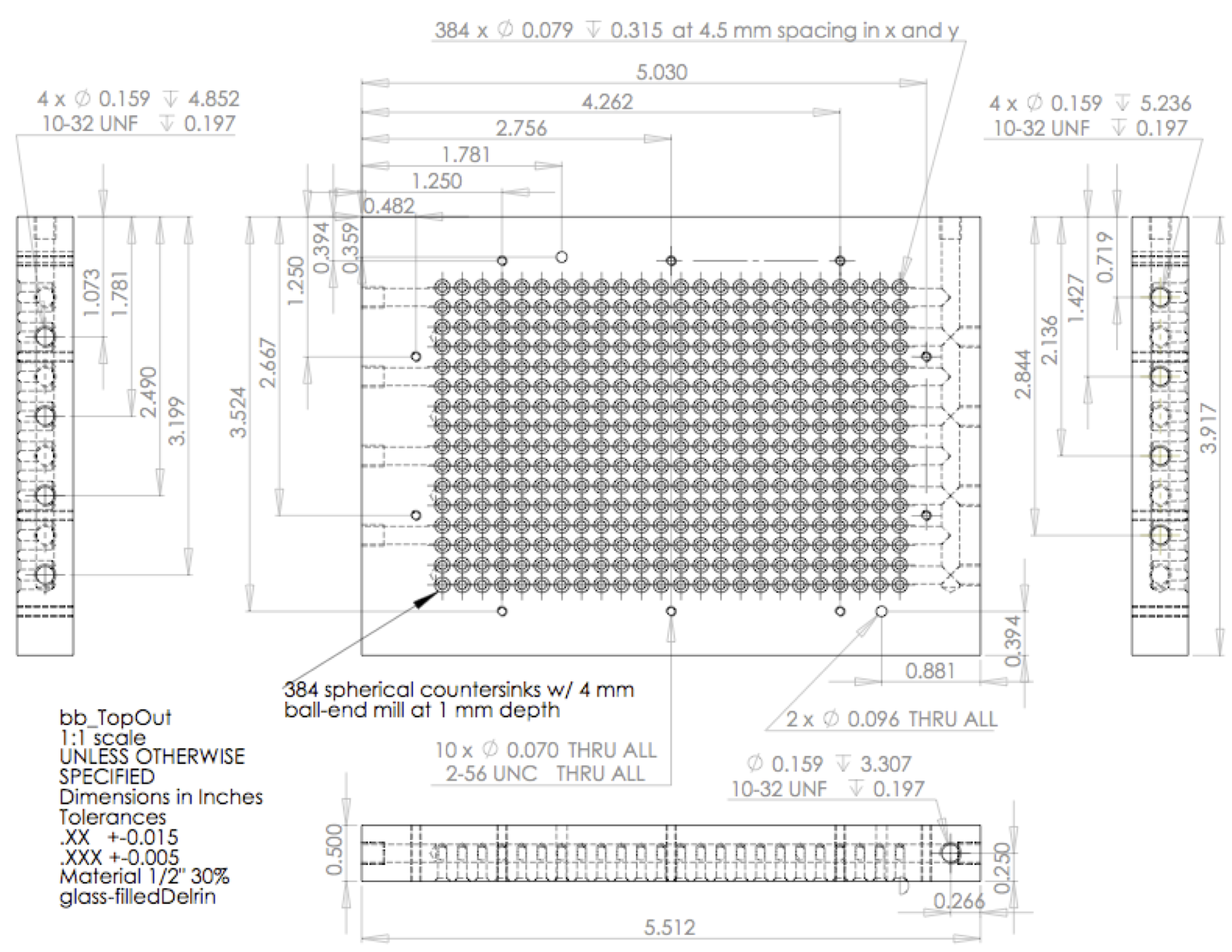

Supplement: Figure S4 — Engineering drawing of outer plate for backtransfer device. (TIFF) [file pone.0032299.s004.tif]

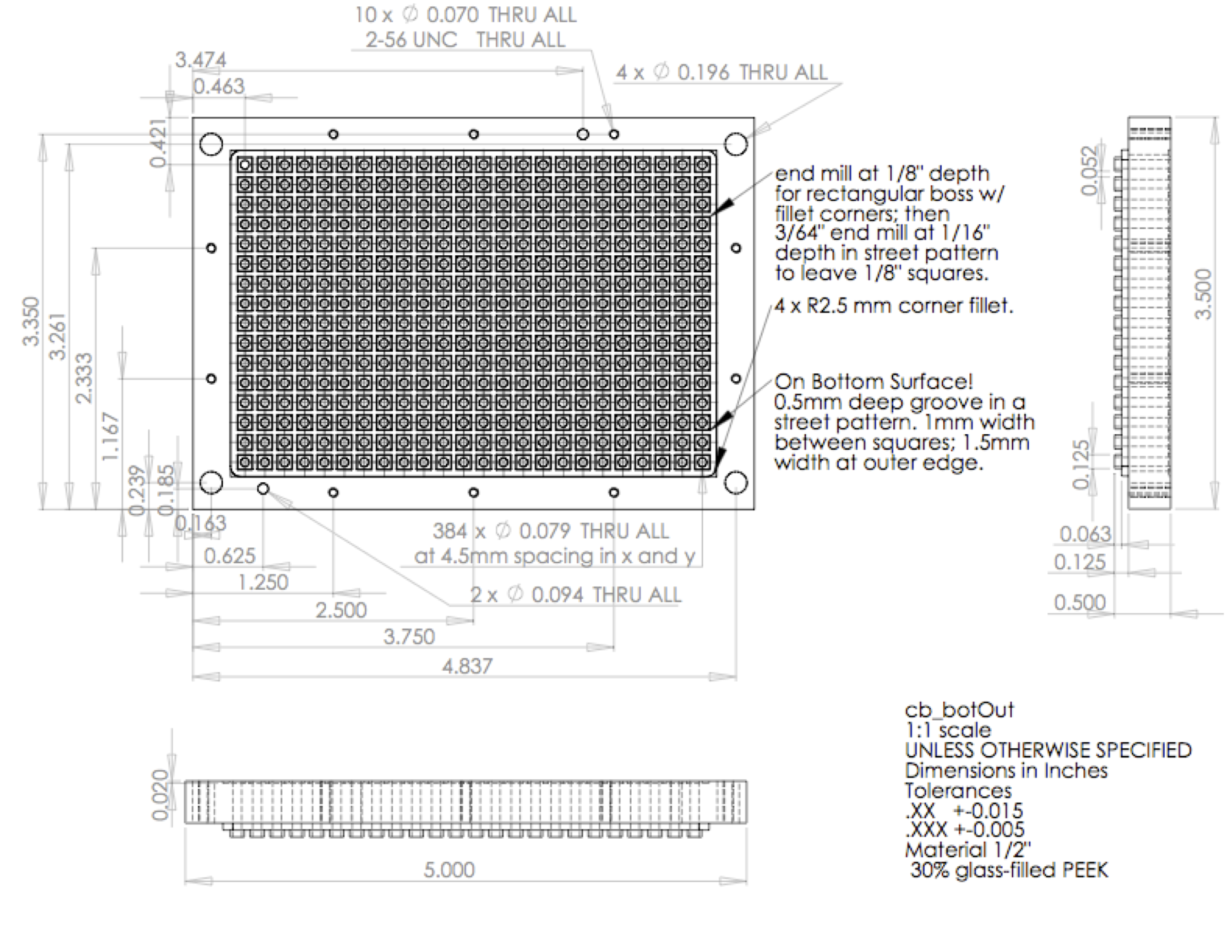

Supplement: Figure S5 — Engineering drawing of bottom plate for chemistry device. (TIFF) [file pone.0032299.s005.tif]

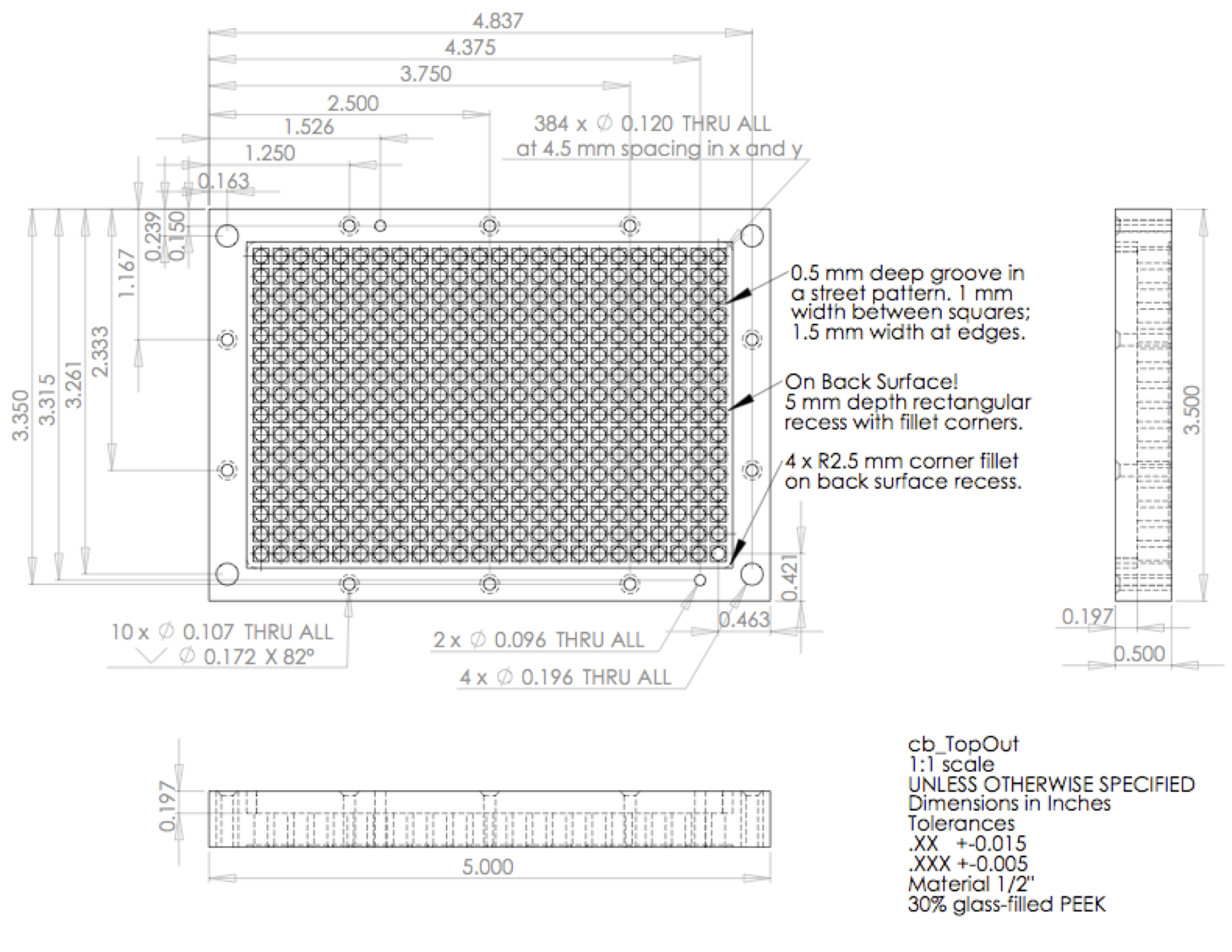

Supplement: Figure S6 — Engineering drawing of top plate for chemistry device. (TIFF) [file pone.0032299.s006.tif]

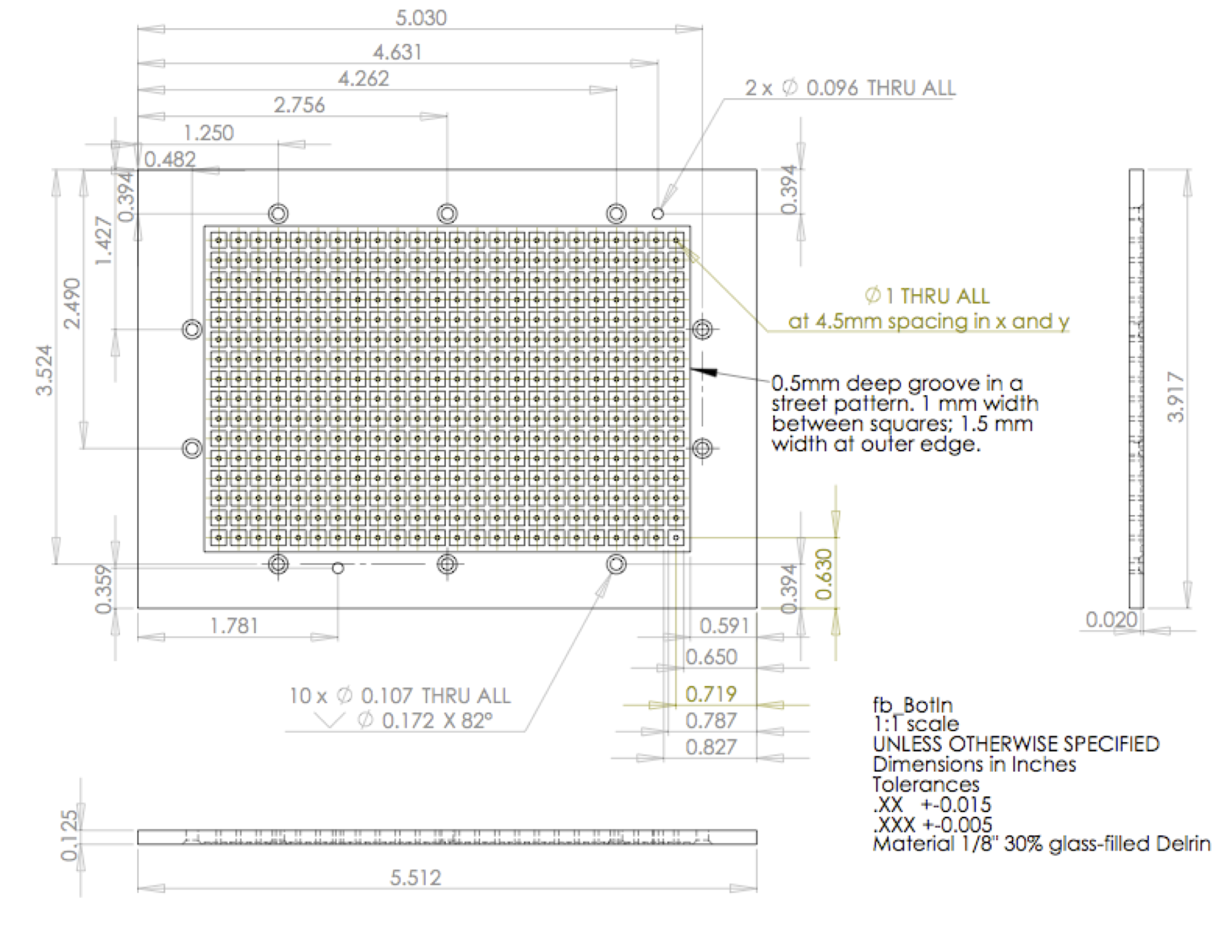

Supplement: Figure S7 — Engineering drawing of internal plate for mesofluidic pump. (TIFF) [file pone.0032299.s007.tif]

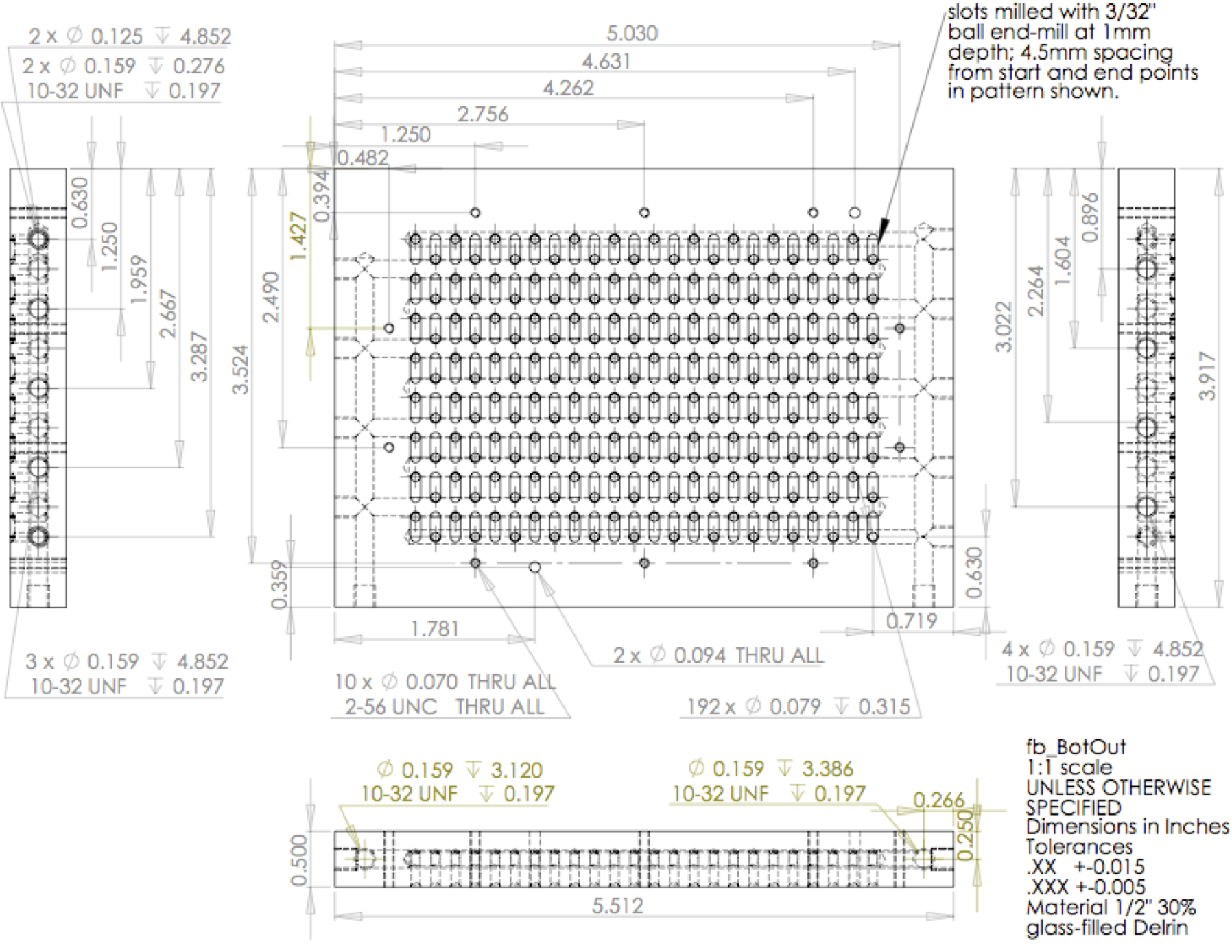

Supplement: Figure S8 — Engineering drawing of outer plate for mesofluidic pump. (TIFF) [file pone.0032299.s008.tif]

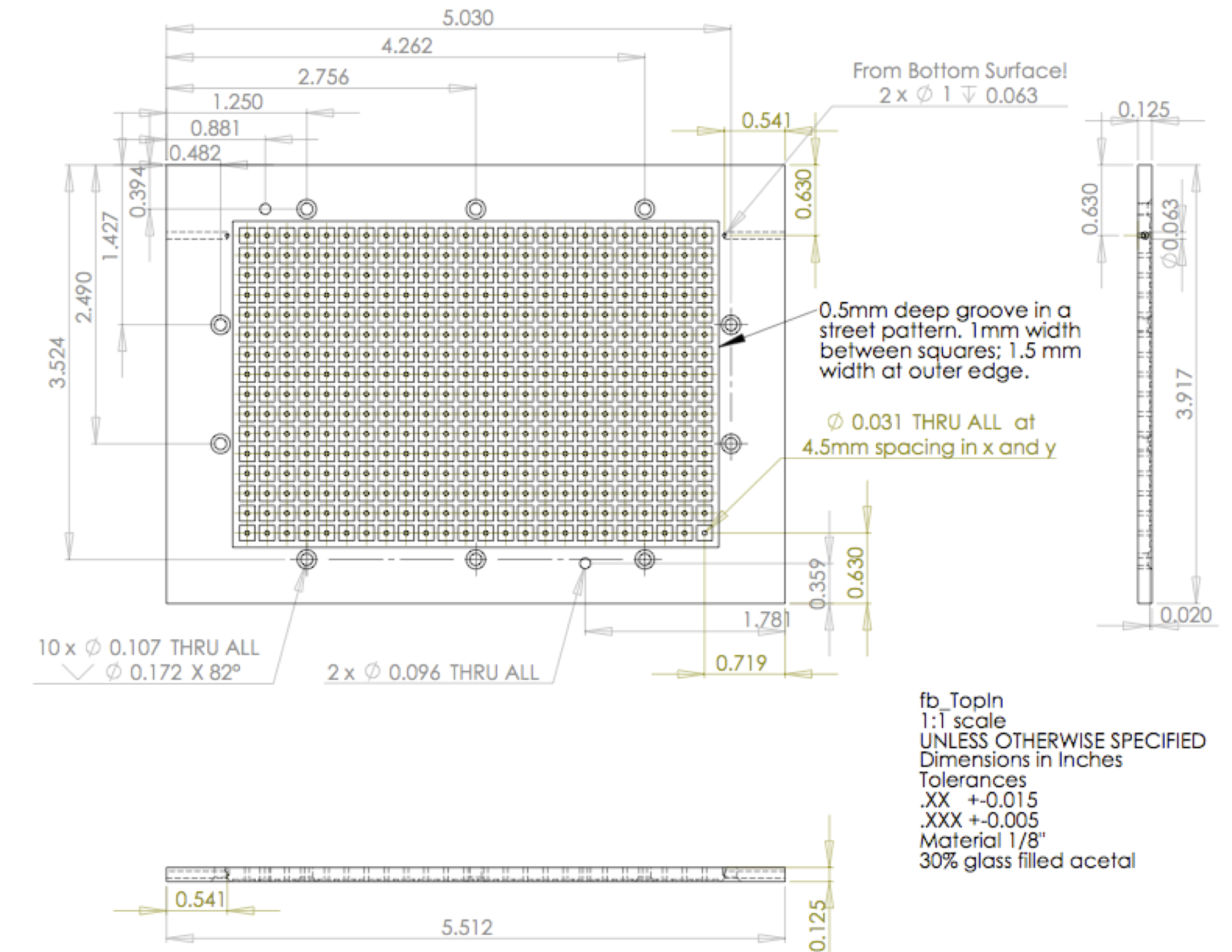

Supplement: Figure S9 — Engineering drawing of internal plate for mesofluidic pump. (TIFF) [file pone.0032299.s009.tif]

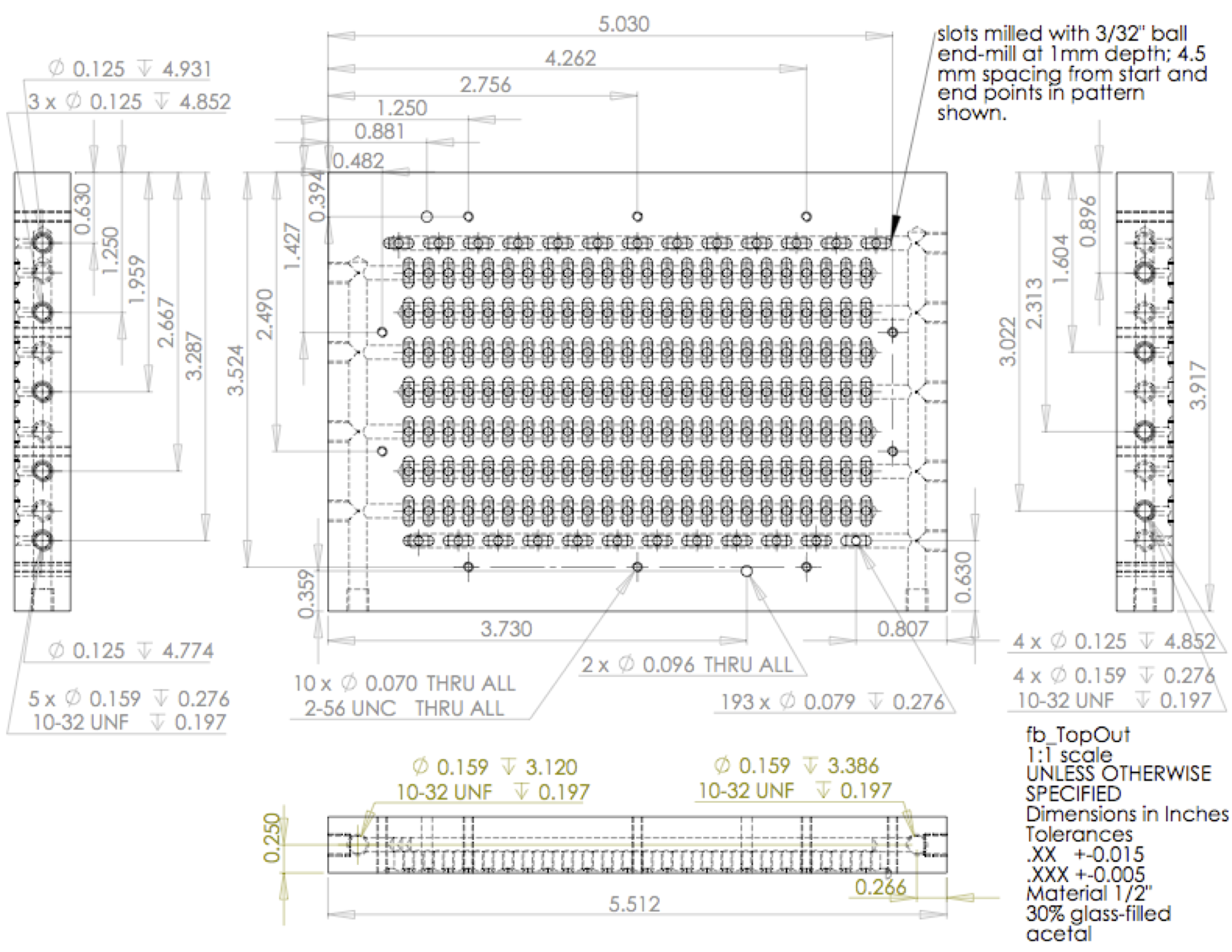

Supplement: Figure S10 — Engineering drawing of outer plate for mesofluidic pump. (TIFF) [file pone.0032299.s010.tif]

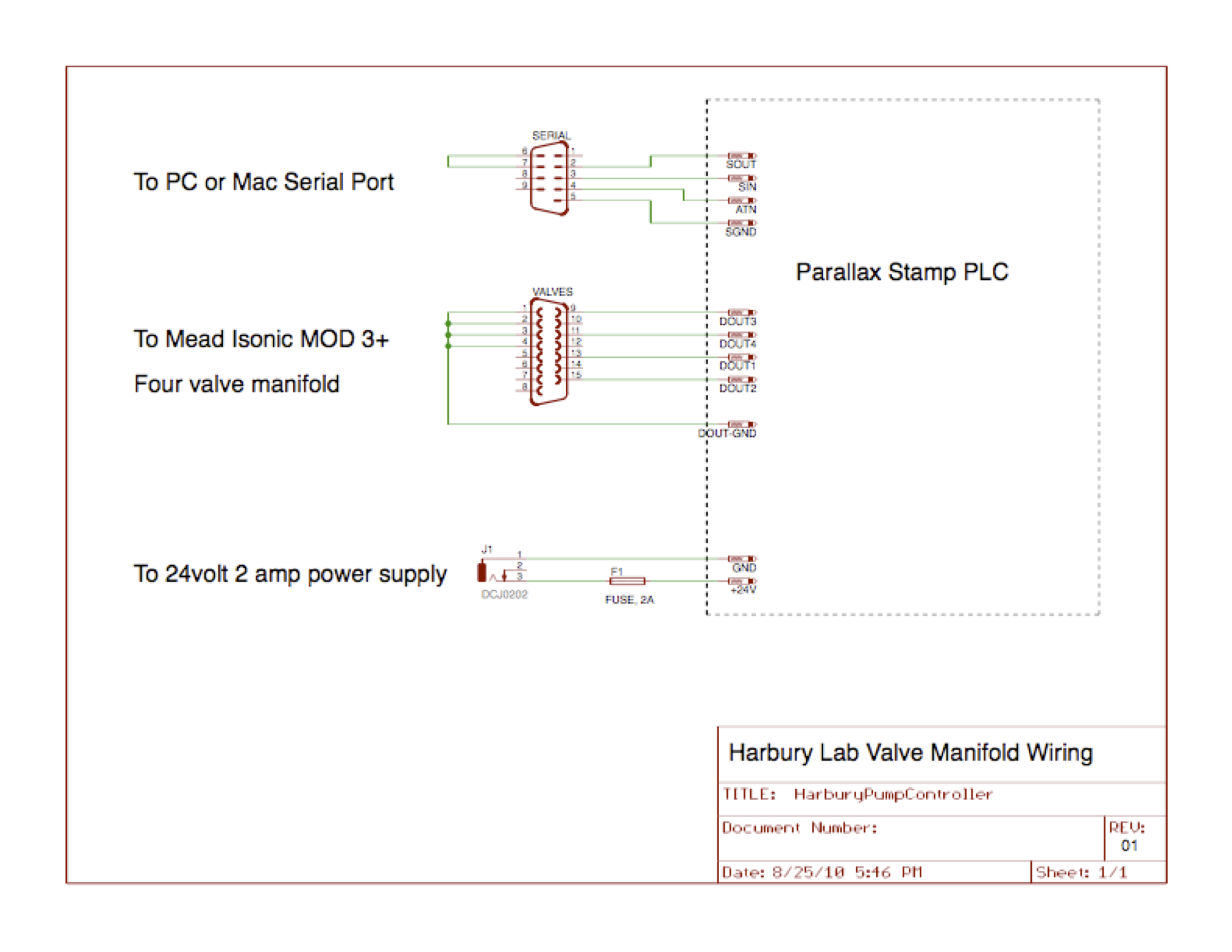

Supplement: Figure S11 — Circuit diagram for Stamp PLC. (TIFF) [file pone.0032299.s011.tif]
