# Supplementary material for: Mesofluidic Devices for DNA-Programmed Combinatorial Chemistry
Source: PLoS One. 2012 Mar 29;7(3):e32299. doi: 10.1371/journal.pone.0032299 (PMC3315586; doi:10.1371/journal.pone.0032299)
Supplement: Table S1 — Masses of peptoid-DNA conjugates in Figure 2b following digestion with nuclease P1. (DOCX) [file pone.0032299.s012.docx]

**Table S1**

| **Alkylation reagent** | **Expected** | **[M-H]^-^** | **[M+H]^+^** |
| --- | --- | --- | --- |
| *Agmatine* | 684.38 | 683.74 | 685.58 |
| *3-amino-1-propanol* | 629.33 | 628.63 | 630.55 |
| *Benzylamine* | 661.34 | 660.67 | 662.55 |
| *ethylene diamine* | 614.33 | 613.66 | 615.50 |
| *2-ethylhexylamine* | 683.41 | 682.79 | 684.69 |
| *ethanolamine* | 615.31 | 614.64 | 616.52 |
| *2-(furfurylthio)ethylamine* | 711.32 | 710.69 | 712.59 |
| *propargylamine* | 609.30 | 608.61 | 610.50 |
| *propylamine* | 613.34 | 612.66 | 614.54 |
| *(R)-(+)-1-phenylethylamine* | 675.35 | 674.68 | 676.63 |
| *trans-4-aminocyclohexanol* | 669.36 | 668.74 | 670.58 |
