# Supplementary material for: Mesofluidic Devices for DNA-Programmed Combinatorial Chemistry
Source: PLoS One. 2012 Mar 29;7(3):e32299. doi: 10.1371/journal.pone.0032299 (PMC3315586; doi:10.1371/journal.pone.0032299)
Supplement: Supporting Information S1 — Parts list and BASIC stamp programs for mesofluidic devices. (DOC) [file pone.0032299.s013.doc]

**Supporting Information S1**

Parts list

To assemble the mesofluidic devices, a collection of small parts and bulk materials are necessary in addition to the machined plates. The following is a list of these items and their catalogue numbers:

Small parts:

- 30 2-56 18-8 slotted fillister and flat-head screws (McMaster-Carr #91781A085, #91794A080, and #91794A081)

- 4 10-32 nylon wing nuts (McMaster-Carr #94924A012)

- 4 10-32 nylon machine screws (McMaster-Carr #94607A838)

- 6 push-to-connect tube fittings (McMaster-Carr #52065K111)

- 2 nylon plugs (McMaster-Carr #51025K251)

- 6 stainless steel dowel pins (McMaster-Carr #90145A445)

- 4 3/8”-16 x 2.5” socket cap screws (McMaster-Carr #92196A634)

Bulk materials:

- Silicone or fluorosilicone rubber sheet (McMaster-Carr #3788T22, #8632K412 or #2183T12)

- Silicone rubber film (McMaster-Carr #86435K43)

- Rubber foam sheet (McMaster-Carr #8785K842)

- Nylon tubing in assorted colors (McMaster-Carr #5548K46)

- 1/16” OD PEEK tubing (Fisher Scientific #05-701-5)

BASIC stamp programs for hybridization and backtransfer

**hyb.bs2:**

'{$STAMP BS2}

'{$PBASIC 2.0}

valve **VAR** **BYTE**(8) 'array of valve states

pump **VAR** **BYTE**(8) 'array of pump states

delay **VAR** **BYTE**(8) 'array of time (in ms) to stay in state

n **VAR** **BYTE** ' counter 0...7

long **CON** 75 ' 1/100th seconds each valve closed

short **CON** 10 ' 1/100th seconds valve overlap

closed **CON** 0

open **CON** 1

valve(0) = (open*1) + (closed*2) + (closed*4) + (closed*8)

valve(1) = (open*1) + (open*2) + (closed*4) + (closed*8)

valve(2) = (closed*1) + (open*2) + (closed*4) + (closed*8)

valve(3) = (closed*1) + (open*2) + (open*4) + (closed*8)

valve(4) = (closed*1) + (closed*2) + (open*4) + (closed*8)

valve(5) = (closed*1) + (closed*2) + (open*4) + (open*8)

valve(6) = (closed*1) + (closed*2) + (closed*4) + (open*8)

valve(7) = (open*1) + (closed*2) + (closed*4) + (open*8)

delay(0) = long

delay(1) = short

delay(2) = long

delay(3) = short

delay(4) = long

delay(5) = short

delay(6) = long

delay(7) = short

**DIRS** = $ffff ' set all I/O pins to output

**GOTO** main_loop

**main_loop:**

**FOR** n = 0 **TO** 7

**OUTD** = valve(n)

**PAUSE** delay(n)

**NEXT**

**GOTO** main_loop

**END**

**backtransfer.bs2:**

'{$STAMP BS2}

'{$PBASIC 2.0}

valve **VAR** **BYTE**(8) 'array of valve states

pump **VAR** **BYTE**(8) 'array of pump states

delay **VAR** **BYTE**(8) 'array of time (in ms) to stay in state

n **VAR** **BYTE** ' counter 0...7

long **CON** 250 ' 1/100th seconds each valve closed

short **CON** 500 ' 1/100th seconds valve overlap

closed **CON** 0

open **CON** 1

valve(0) = (open*8) + (open*4) + (open*2) + (closed*1)

valve(1) = (open*8) + (open*4) + (closed*2) + (open*1)

delay(0) = long

delay(1) = long

**DIRS** = $ffff ' set all I/O pins to output

**GOTO** main_loop

**main_loop:**

**FOR** n = 0 **TO** 1

**OUTD** = valve(n)

**PAUSE** delay(n)

**NEXT**

**GOTO** main_loop

**END**
